# Lifestyle intervention in children with obesity and nonalcoholic fatty liver disease (NAFLD): study protocol for a randomized controlled trial in Ningbo city (the SCIENT study)

**DOI:** 10.1186/s13063-024-08046-4

**Published:** 2024-03-20

**Authors:** Ping-ping Zhang, You-xin Wang, Fang-jing Shen, Yun-fei Xing, Jia-ying Gu, Xue-ying Li, Han Jin, Shi-feng Jin, Miao Xu, Hai-jun Wang, Hui Wang, Li Li

**Affiliations:** 1 Ningbo Center for Healthy Lifestyle Research, The First Affiliated Hospital of Ningbo University, Ningbo, Zhejiang Province 315000 China; 2 Department of Maternal and Child Health, School of Public Health, Peking University, Beijing, 100191 China; 3 Department of Endocrinology and Metabolism, The First Affiliated Hospital of Ningbo University, Ningbo, Zhejiang Province 315000 China

**Keywords:** Children, Obesity, NAFLD, Lifestyle intervention, M-Health

## Abstract

**Background:**

The increasing prevalence of childhood obesity has become an urgent public health problem, evidence showed that intervention for childhood obesity bring enormous health benefits. However, an effective individualized intervention strategy remains to be developed, and the accompanying remission of related complications, such as nonalcoholic fatty liver disease (NAFLD), needs to be assessed. This study aimed to develop an m-Health-assisted lifestyle intervention program targeting overweight/obese children and assess its effectiveness on indicators of adiposity and NAFLD.

**Methods:**

This is a cluster-randomized controlled trial that conducted in children with overweight/obesity in Ningbo city, Zhejiang Province, China. Students in Grade 3 (8–10 years old) were recruited from six primary schools, with three be randomized to intervention group and three to usual practice group. The intervention program will last for one academic year and consists of health education, dietary guidance, and physical activity reinforcement. This program is characterized by encouraging four stakeholders, including School, Clinic, famIly, and studENT (SCIENT), to participate in controlling childhood obesity, assisted by m-Health technology. Assessments will be conducted at baseline and 3 months, 9 months, 24 months, and 36 months after baseline. The primary outcome will be the differences between the two groups in students’ body mass index and fatty liver index at the end of the intervention (9 months after baseline). During the implementation process, quality control methods will be adopted.

**Discussion:**

The program will test the effectiveness of the m-Health-assisted lifestyle intervention on children with obesity and NAFLD. The results of this study will provide evidence for establishing effective lifestyle intervention strategy aimed at childhood obesity and NAFLD and may help develop guidelines for the treatment of obesity and NAFLD in Chinese children.

**Trial registration:**

Clinicaltrials.gov NCT05482191. Registered on July 2022.

**Supplementary Information:**

The online version contains supplementary material available at 10.1186/s13063-024-08046-4.

## Introduction

The increasing prevalence of childhood obesity has become an urgent public health problem worldwide, arising as a common concern of the country, schools, families, and other sectors of society [1, 2]. The China Chronic Disease and Nutrition Surveillance 2015–2019 showed that the prevalence of overweight and obesity in 6- to 17-year-old children was 11.1% and 7.9%, respectively [3]. The COVID-19 pandemic enhanced the sedentary lifestyle due to home lockdown [4, 5]. Obesity that occurs in childhood tends to persist into adulthood, predisposing individuals to both physical and psychosocial health consequences in the short and long term, such as hypertension, dyslipidemia, nonalcoholic fatty liver disease (NAFLD), diabetes, and cardiovascular disease [1]. In addition, children with obesity are more likely to have social and psychological problems such as bullying, self-esteem, depression, and anxiety [1]. It is meaningful to conduct interventions to protect them from obesity-related adverse health consequences. Evidence has shown that effective interventions for childhood obesity has enormous dividends [6], highlighting the urgent need to develop effective interventions.

Previous research indicated that multicomponent strategies that combined physical activity, nutritional, and educational interventions were demonstrated to have better outcomes than single-component strategies in the past practice [7–9]. However, in traditional Chinese culture, children are encouraged to “eat more to be healthy” [10]. As parents do not consider childhood obesity as an unhealthy condition, the willingness to help their children with a healthy weight management is not strong. Obese people have a much stronger willingness to lose their weight when the doctor tells them that they have an obesity-related disease, for example, NAFLD. NAFLD was screened with FibroScan Handy (Echosens, Paris, France) to alter the severity of obesity among children [11]. Food consumption patterns in China are complicated, over- and under-nutrition coexist, and the intake of cereals, oils, and salt are excessive, with moderate-to-serious deficits in the intake of vegetables, fruits, fish, milk, and soybeans [12, 13]. The latest “Dietary Guidelines for Chinese Residents (2022)” for children emphasize improving students’ nutritional literacy and advocate for families to build a healthy food environment. However, parents of obese children lack knowledge of selecting and making healthy food, and they need knowledge from professional nutritionists to guide the intake of foods.

Thus, the present study combines the effects of schools, clinics, families, and students to build a healthy lifestyle environment for school-aged children since they can build a healthy lifestyle easily and benefit from long-term effects. With the development of mobile health (m-Health) in prevention obesity methods, m-Health and artificial intelligence technology could build up the real-time connection between school–clinical–family–students via a positive-feedback loop through measurements–evaluation–intervention–measurements and further enhance healthy behaviours among children [14]. However, current research on the effectiveness of m-Health-assisted intervention on childhood obesity still has conflicts [15].

Compared with previous studies, the present study will take advantage of experts from clinic, such as nutritionists and ultrasound technician to set up the authority of present study. With the aid of clinic, help each overweight/obesity student and their parents gain the knowledge of healthy food selecting, food making, and the severity of obesity for each student. In addition, using the m-Health to connect school–clinic–family–students and strength the compliance of intervention. Our current interventional design could provide evidence on the effect of a multifaced (School–Clinic–famIly–studENT, SCIENT) lifestyle intervention on childhood overweight/obesity/NAFLD. Additionally, providing evidence of the effects on the m-Health in childhood intervention program.

### Aims of this trial

The aims of the SCIENT study were to conduct a lifestyle intervention for childhood obesity to: (1) evaluate the effectiveness of the lifestyle intervention compared with the usual practice in treating childhood overweight and obesity, and (2) evaluate whether interventions for weight loss would improve obesity-related liver features in overweight and obese children.

## Methods

### Study design

**Fig. 1:**
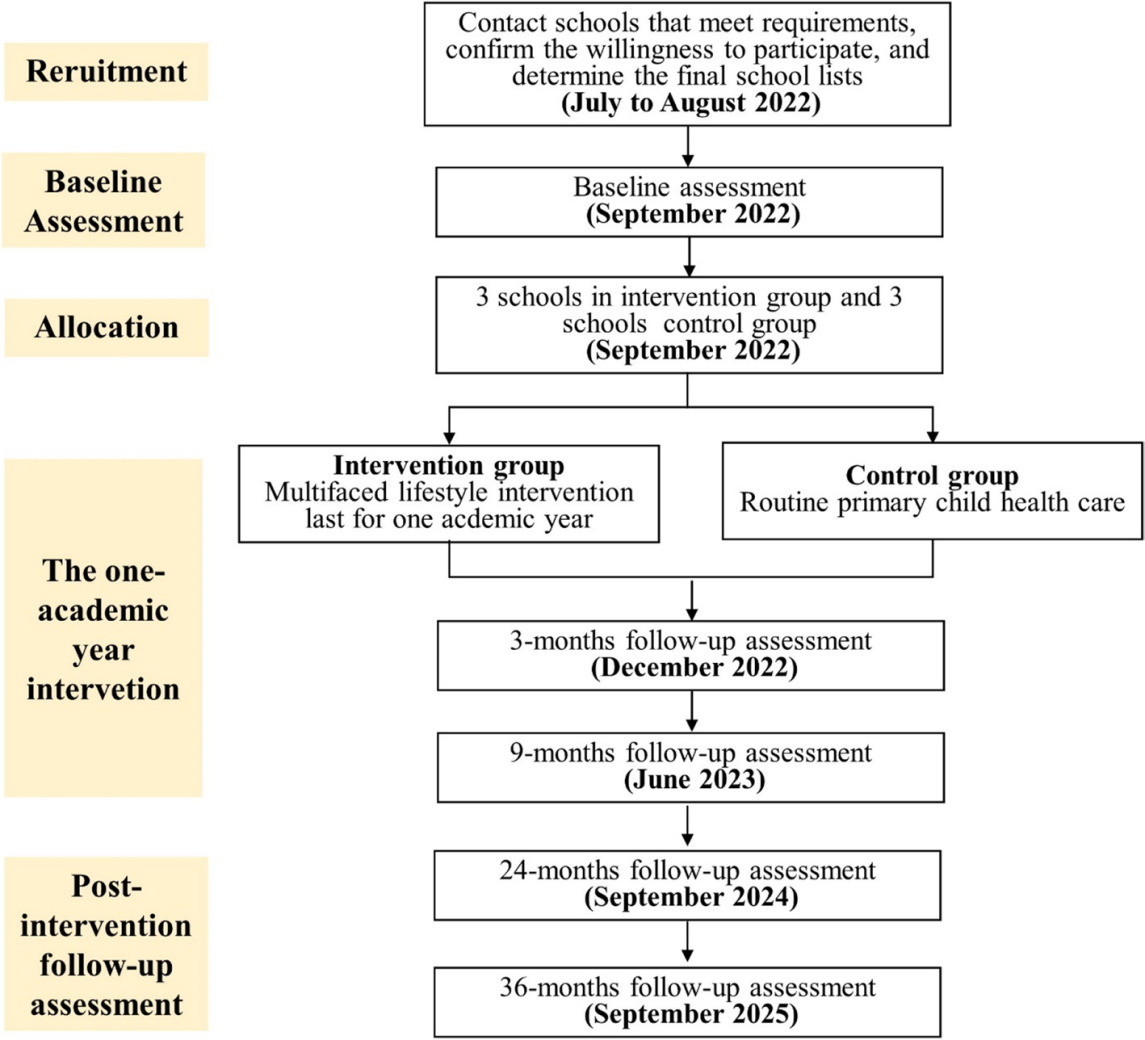
Study design of the trial

The study will be a cluster-randomized controlled trial carried out in six primary schools in Ningbo, one of the most socioeconomically advanced cities in southeast China. The allocation of schools to the intervention or control group will be performed by random number, and the randomization will be conducted after baseline assessments to ensure the concealment of the allocation. Given the features of the intervention, blinding students or teachers at the participating schools was not allowed. All students diagnosed with overweight/obesity in Grade 3 at baseline will be invited to take part in our program, with informed consent by all participating students and their primary guardians. Study design of this trial was shown in Fig. 1.

### Recruitment

#### Schools

The recruitment of the participating schools followed two steps: (1) contact local education authorities for cooperation and support and collect the basic information of the recommended schools, and (2) contact and visit the schools to confirm the eligibility and willingness to participate and determine the list of participating schools.

This project will be conducted in Grade 3 students (aged 8 to 10 years old), as their comprehension is sufficient to understand the whole program. Participating schools will be selected based on the following criteria: school managers being interested in and agreeing to implement the project; having project-related staff (such as school doctors/health care physicians, physical education teachers, student canteen nutritionists); not boarding schools; not specialty schools for children with talents or minority ethnicities; and not having or planning to implement a similar intervention project.

#### Participants

After finishing the national annual routine physical examination at the beginning of the academic year, a presentation of the project will be held for all caregivers (parents in most cases) eligible for overweight and obese children in each school to invite participants. Written informed consent will be signed by all participating students and their primary guardians. The inclusion criteria for students were as follows: students in Grade 3 aged 8 to 10 years old and students with childhood overweight/obesity defined according to the criteria for Chinese children and adolescents [16]. Students with the following conditions will be excluded: a medical history of heart disease, hypertension, diabetes, asthma, viral hepatitis or nephritis; obesity caused by endocrine diseases or drugs; abnormal physical development or physical deformity; inability to participate in school sport activities; and a loss in weight by vomiting or taking drugs during the past 3 months.

### Randomization and blinding

The randomization of the schools (clusters) to the intervention or control arm will be allocated using a computer-generated random number system (the simple random sampling method). Randomization is carried out after baseline assessment, and then all participating schools are informed of the allocation grouping. Table 1 provides an overview of the study process.

**Table 1:**
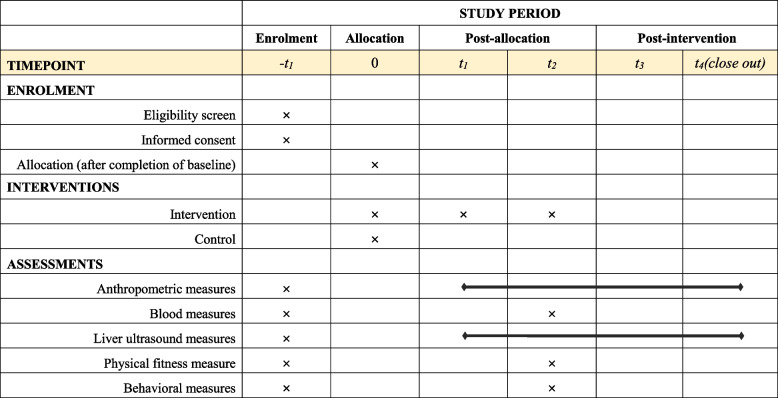
Participant timeline according to SPIRIT

t_1_: 3 months after baseline; t_2_: 9 months after baseline; t_3_: 24 months after baseline; t_4_: 36 months after baseline

### Intervention

The SCIENT study was based on socioecological model theory [17] and comprised activities aimed at helping children build healthy dietary behaviours and increasing physical activities in school and at home. This lifestyle intervention program focused on four components. School-based lifestyle interventions will receive support from clinics (specialist doctors and nutritionists) and family members, followed by uniform implementation manuals. All school staff will receive reimbursement to make up for their extra work during the intervention. Parental involvement is an important feature in this study. A trained professional nutritionist is allocated to each student and his/her parents to help with individualized dietary instruction, and an official account and chat group on WeChat are used as the platform for knowledge information dissemination.

#### Key components of intervention

**Fig. 2:**
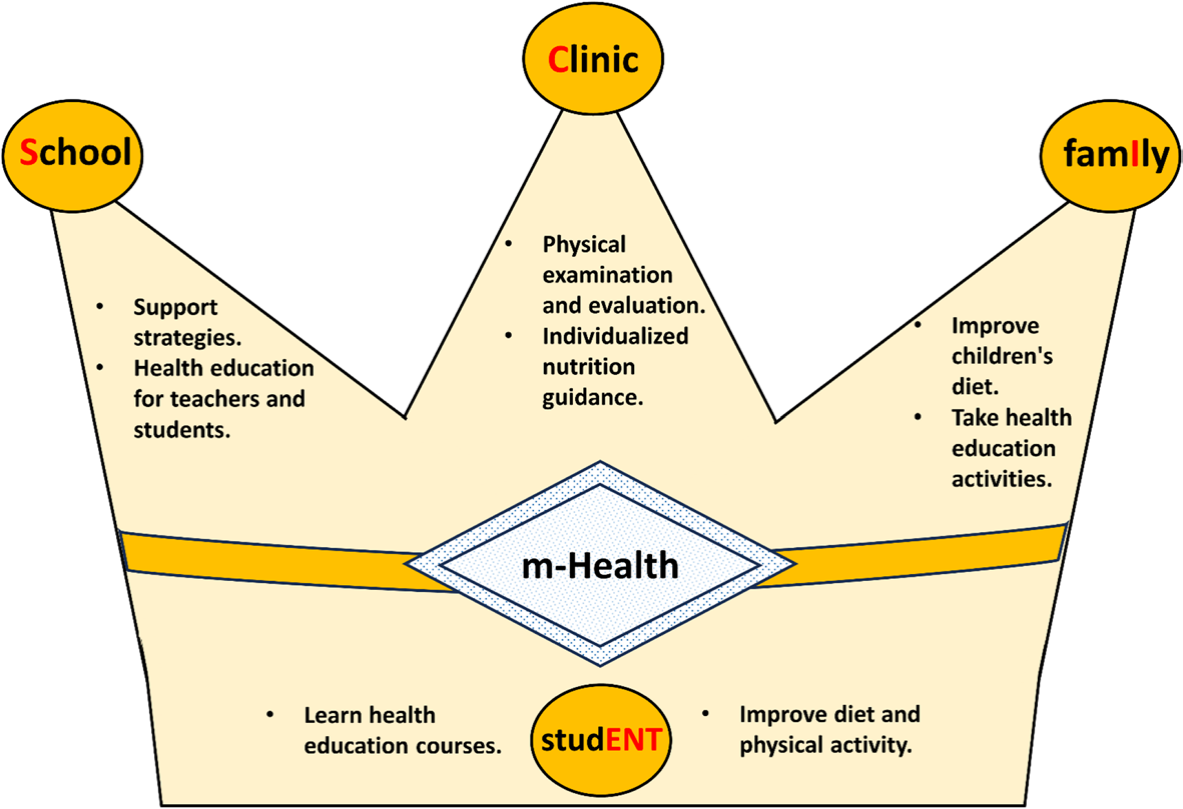
Framework of the SCIENT intervention program

This is a multicomponent intervention program containing four intervention components and involving four stakeholders, including school, clinic, family, and student, assisted by m-Health technology. Each component has fixed key items and elements, and the framework of this SCIENT intervention program is shown in Fig. 2.

### Component 1: Carry out health education courses and distribute health education materials

Ten health education courses that were specially designed for primary school students will be released biweekly by trained class teachers, with six courses arranged in the first semester and four in the second semester. The core messages (2–2-1 core messages: 2 not: not eating excessively, not drinking sugar-sweetened beverages; 2 less: less high-energy food, less sedentary time; 1 more: more physical activities) of the courses come from our previous DECIDE-Children study [18], which mainly include the benefits of healthy weight, measurements and assessments of weight, and methods of achieving a healthy weight.

We will organize two health education activities for parents. The two health education activities with key messages similar to health education courses for students will be delivered at the beginning of each semester, and each activity will last for approximately 50 - 60 min. During the first activity, the baseline physical examination report will be fed back to parents, and an online WeChat group with approximately 30 caregivers will be established with one nutritionist for further Q&A. For the second activities, project staff will provide feedback about the student’s weight management status and answer parents’ questions about students’ weight management face-to-face.

The materials of a healthy lifestyle for weight management education will be distributed in various ways. Posters that contain weight management information released in ten courses will be put up in the classroom in intervention schools during the program. Health education books designed for elementary school students and “nutrition evaluation turnplate for Chinese primary and middle school students” [18] will be delivered to students in the intervention group. Moreover, mental health books designed for school-age students will also be distributed. Popular science articles and cartoon videos and cases with good performance will irregularly be released on our official WeChat account to intensify the consciousness of a healthy lifestyle of the students and primary caregivers.

### Component 2: Nutrition guidance by professional nutritionists

Students will receive individual dietary guidance from an assigned professional nutritionist when they eat at home or eat school lunch and afternoon tea at school. Professional nutritionists will help caregivers to improve the dietary pattern at home. A 21-day lifestyle intensive nutrition intervention at the beginning of the first semester and a 7-day lifestyle intensive nutrition intervention at the beginning of the second semester will be provided by nutritionists through our official WeChat account. Then, the nutritionists will provide nutrition guidance at least once a week during each semester. Caregivers upload the food pictures that the participating children eat on the scheduled days, and nutritionists will give individualized advice about how to improve diet at home, taking into account their own diet characteristics. The initial goal of dietary approaches includes increasing the intake of vegetables and fruit, reducing foods with high energy density but poor nutrients, avoiding sugar-sweetened beverages, eating breakfast, appropriate portion size, and reducing eating outside [19].

Moreover, nutritionists will also help canteen staff modify school lunch and afternoon tea when children eat at school. For students in the intervention groups, healthy lunches will be prepared by dedicated canteen staff. In addition to reducing total energy, some unhealthy foods will be replaced with foods better for weight loss, for example, replacing refined rice with roughage rice, reducing foods high in oil and fat and increasing the proportion of vegetables and fruits, and replacing sweetened yogurt with sugar-free yogurt.

### Component 3: Reinforce physical activity

Different physical activities in/outside school will be encouraged to improve the intensity and duration of physical activity in both school and home settings. According to the Chinese national requirement for “One-Hour Physical Activity on Schoolyard Every School Day”, schools will ensure that all students have at least 1 h of moderate-to-vigorous intensity activity during school hours. In addition, for participants in our program, a full-time PE teacher helped organize at least 30 min of exercise after class on school days three times a week, mainly focusing on moderate- to high-intensity aerobic exercise supplemented by anaerobic exercise. Any other form of exercise with family members and partners outside school will also be encouraged.

Each student in the intervention schools will receive an Amazfit Neo sports watch (Zepp Health, Beijing, China), which can record exercise, heart rate, sleep conditions, etc. Caregivers can check the student’s exercise and sleep conditions through an App linked with the watch, which will be helpful for improving the physical activity level of students at home.

### Component 4: Develop and implement school policies related to weight loss

School behavioural support strategies related to obesity prevention and management are formulated and implemented in intervention schools, contributing to healthy lifestyle improvement, including both balance diet and fitting exercise. To optimize the dietary environment in school, the school will: (1) integrate health and nutrition education into school curricula and teaching plans; and (2) create a healthy school eating environment by banning foods high in fat, salt, and sugar from school canteens, supermarkets, and vending machines. For optimizing the exercise environment in school, the school will: (1) ensure that students have enough time for physical activity; and (2) build an environment for physical activity and increase places and facilities for physical exercise.

Health education activities for school teachers and canteen staff will be released. The training activities of the teacher will be delivered at the beginning of each semester, which will last for approximately 50 - 60 min and mainly focus on the key messages of health education courses and how to deliver the courses according to the prepared lesson plan. The nutritionist will hold face-to-face nutrition coaching with canteen staff in each intervention school on the basis of providing nutritionally complete foods to increase the intake of vegetables and fruit and reduce energy-dense nutrient-poor foods.

#### Apply m-Health technology

WeChat is the most popular instant messaging App in China. In this study, we referred to the functions of the “Eat Wisely, Move Happily” App used in the DECIDE-Children study [18] and realized the functions of the App to the WeChat public account “Population Health Service Platform”, including information diffusion, behaviour monitoring, weight management, assessment, and feedback. Moreover, we added a new function for dietary management, which required the participants to upload pictures of all foods, their main meals and extra meals, and the corresponding nutritionist provided individualized suggestions for dietary optimization.

We will also set up parents’ WeChat groups in each intervention school, each contains approximately 30 families, to deliver healthy eating knowledge and provide support. Wearable devices for improving health care processes will be applied as well. Each student in the intervention schools will receive an Amazfit Neo sports watch to help caregivers check the student’s goal attainment of daily exercise and sleep.

#### Quality control of the intervention

We took a series of methods to ensure that the intervention was implemented as planned. Two comprehensive operation manuals were developed—one for project staff and another for school team members—to guide the execution and management of this multifaceted intervention. To enhance caregiver involvement, we provide reminders through WeChat messages.

### Control group

During the trial, the three schools in the control group will continue their original teaching plan and will not receive any components conducted in the intervention group. After the intervention, all activity resources used in the intervention group will be delivered to the control group.

### Data collection and management

Assessments of the participants will be undertaken in school by trained research staff. The data we collected include anthropometric, blood, liver ultrasound, physical fitness, and behavioural measures, and the detailed variables and measuring tools are described in Table 2. The baseline assessments will be conducted in September 2022, and all participants from intervention and control schools will accept these assessments. Follow-up assessments will be conducted 3, 9, 24, and 36 months after baseline. All data will be managed by designated personnel with paper-based study data held in locked filing cabinets.

**Table 2:** Outcome assessment

Variables	Measuring methods	Baseline (2022.9)	3 months after baseline (2022.12)	9 months after baseline (2023.6)	24 months after baseline (2024.9)	36 months after baseline (2025.9)
Anthropometric measures						
Height	Stadiometer	√	√	√	√	√
Weight	Lever scale	√	√	√	√	√
Waist circumference	Tape	√	√	√	√	√
Hip circumference	Tape	√	√	√		
Blood pressures	Digital sphygmomanometer	√	√	√		
Body composition	Inbody770 (Biospace, USA)	√	√	√		
Blood measures						
Fasting glucose	Follow clinical standard protocols	√		√		
Fasting insulin		√		√		
Triacylglycerol		√		√		
Total cholesterol		√		√		
Low-density lipoprotein cholesterol		√		√		
High-density lipoprotein cholesterol		√		√		
Alanine aminotransferase		√		√	√	√
Liver ultrasound measures						
Liver stiffness measurement	FibroScan HANDY (Echosense, France)	√	√	√	√	√
Controlled attenuation parameters	FibroScan HANDY (Echosense, France)	√	√	√	√	√
Physical fitness measure						
20-m shuttle-run test	Conducted by trained physical health teacher	√		√	√	√
Behavioural measures						
Students’ eating behaviour	Dietary quality questionnaire (DQQ) [20]	√		√		
Students’ physical activity behaviour	Self-designed questionnaire [18]	√		√		
Psychological status	Center for Epidemiologic Studies Depression Scale (CES-D Scale) [21] and Social Anxiety Scale for children (SASC) [22]	√		√		
Sleep quality	Pittsburgh sleep quality index (PSQI) [23]	√		√		

### Outcomes

For evaluating the effectiveness of intervention, the primary outcomes include differences between intervention and control groups in the changes after one academic year intervention (9 months after the baseline) of three indexes: (a) BMI (calculated as the individual’s body weight in kilograms divided by the square of body height in meters), (b) controlled attenuation parameters (CAP, detected by FibroScan HANDY, Echosens, Paris, France), and (c) liver stiffness measurement (LSM, detected by FibroScan HANDY, Echosens, Paris, France). Secondary outcomes include differences of other measures between intervention and control groups in the changes after one academic year intervention: (a) changes in BMI Z score (SD score will be calculated based on the WHO criteria [24]), (b) changes in blood glucose, (c) changes in blood lipids (including triacylglycerol (TG), total cholesterol (TC), low-density lipoprotein cholesterol (LDL-c), and high-density lipoprotein cholesterol (HDL-c)), (d) changes in fasting insulin, (e) changes in body fat percentage, (f) changes in waist circumference, (g) changes in cardiorespiratory endurance test, (h) changes in alanine transaminase (ALT), and (i) changes in eating behaviour, physical activity behaviour, psychological status, sleep quality, etc. After the intervention finished, we will investigate anthropometric measures and fatty liver indexes in 24 and 36 months after baseline to evaluate the long-term effects of the intervention.

### Sample size

The primary outcome measures in our study included change in BMI, change in CAP, and changes in LSM. According to previous studies, the difference between the two groups in the change of BMI (effect size) was assumed to be 0.87 kg/m^2^ [15], with a standard deviation (SD) of 0.9 kg/m^2^; meanwhile, the changes in CAP and LSM were assumed to be 72 dB/m (with an SD of 75 dB/m) and 0.30 kPa (with an SD of 0.31 kPa) [25], respectively. The intracluster correlation coefficient (ICC) was 0.05, and the rate of attribution was 10% in our study. We aimed to recruit 300 students in total from 6 schools with an average of 50 students in each school. This sample size will provide power of 89.1%, 87.6%, and 88.0% with *α* = 0.05 to detect the set effect size of the change in BMI, CAP, and LSM. All power calculations were carried out in PASS 15.0.5 using the cluster-randomized function.

### Statistical analyses plan

Statistical analyses will be performed using IBM SPSS 26.0 (IBM Inc) and R 4.3.1 (R Core Team), and all two-sided *P* values of 0.05 will be considered significant. The baseline characteristics at the participant level (including anthropometric measures, blood indexes, liver ultrasound measures, and questionnaire survey indexes) and school level (school size, etc.) will be described by intervention and control arms, with normally distributed continuous data described by the mean (standard deviation) and skewed distributed continuous data described by the median (quantiles).

The intention-to-treat analysis will be applied in the analysis of the outcome for all follow-up stages. Generalized linear mixed models will be used to compare the primary and secondary outcomes at 3 and 9 months after the baseline assessment to investigate the effectiveness. Important factors associated with obesity will be adjusted, including baseline outcome values and sex. Outcomes are either binary or continuous, in that either log or linear regression functions will be used, and transformations will be done for any skewed distributed data.

In addition, subgroup analysis (e.g., sex, maternal education level, baseline BMI status), sensitivity analysis (e.g., defined overweight/obesity using the Chinese reference in main analyses and the WHO reference, before and after imputation missing data), and interactive analysis (e.g., whether the intervention effects varied by subgroup) will be explored.

### Health economic evaluation

Health economics evaluation will be conducted by cost-effectiveness analysis, and a societal perspective will be used to examine whether the intervention is economically feasible. Intervention costs will cover the time for all the intervention activities spent by project staff, school staff, and all material expenses. Regarding the time spent implementing the intervention, only that spent by the project staff will be included. Time costs will be based on personal employment compensation (if available) or local average compensation for similar types of employees. Material expenses will be based on the actual purchase prices. An incremental cost-effectiveness ratio will be calculated, and a sensitivity analysis will be used to vary key parameters to check the robustness of the health economics results.

### Patient and public involvement

The patients and key stakeholders (parents, teachers, school principals, nutritionists) will be interviewed for refining the intervention approach and will not involve in any other aspects of the study, including idea generation, study design, program implementation, data collection, and analysis and interpretation of the results. The results of this program will be disseminated through publication in peer-reviewed journals, conference presentations, and reports distributed to school staff, students, and parents. The benefits and burdens of the intervention will be evaluated through self-reported questionnaires for children and their primary caregivers at the end of the intervention.

### Composition of the coordinating center, trial steering committee, and data monitoring committee

The coordinating center and trial steering committee was located in the First Affiliated Hospital of Ningbo University. The responsibility included study planning, responsible for trial master file, organisation of steering committee meetings, participants enrolment, randomisation, intervention implementation, and data verification. A data monitoring committee with a semi-annual reporting structure is implemented, and it is independent from the sponsor and competing interests.

### Frequency and plans for auditing trial conduct

Data monitoring will adhere to the Standard Operating Procedures (SOPs) of the internal Clinical Trials Center (affiliated with the First Affiliated Hospital of Ningbo University). Monitors will systematically scrutinize source documents, as necessary, to ascertain the completeness and accuracy of data documented. The trial steering committee convenes at minimum on a monthly basis throughout the trial, while the data monitoring committee meets semi-annually to evaluate trial conduct comprehensively.

### Protocol amendments

All significant protocol amendments will be disseminated to pertinent stakeholders (such as participating paediatric practices and the ethics committee) by the coordinating center, utilizing newsletters or direct contact. This process will be overseen by the trial steering committee.

### Statement and trial status

The standardized, nonpharmacologic weight loss program will be conducted for one academic year, and all participants will be treated identically and standardized during the whole study period. The study was conducted according to the Declaration of Helsinki [26] and approved by the ethics committee of the First Affiliated Hospital of Ningbo University (Approval No.: 2021-R168). All parents and children provided written informed consent for study participation.

This trial started recruitment of participants in September 2022. Baseline assessments were conducted and completed in the last 3 weeks in September 2022. The intervention will last one academic year and end in June 2023. The 3-, 9-, 24-, and 36- month follow-up assessments will be conducted in December 2022, June 2023, September 2024, and September 2025.

## Discussion

Obesity is caused by excessive energy intake and less physical activity, which increases the risk of chronic noncommunicable diseases and significantly contributes to the global burden of disease. It is critical to adopt healthy behaviours in childhood, as it is a crucial period of shaping behavioural habits in adulthood. A multicomponent individualized intervention could help not only improve obesity-related health disorders but also contribute to the sustainable adherence of a healthy lifestyle pattern [27]. Given the multifactorial aetiology of obesity, it also highlights the importance of developing and applying multifaced and m-Health assisted approaches.

Until now, there has been no approved pharmacological option for the treatment of paediatric NAFLD, and the main management in clinical practice is lifestyle modifications for weight loss. Some clinical guidelines recommend screening of children at high risk for NAFLD [28, 29], such as overweight/obese children, as NAFLD may progress to fibrosis and end-stage liver disease, and earlier recognition can help early prevention. Liver ultrasound is a commonly used method in screening for NAFLD, but its utility as a screening tool is unconfirmed [30] because of inadequate sensitivity and specificity, especially in individuals who are severely obese or have advanced fibrosis [31]. As LSM by vibration-controlled transient elastography (VCTE) and CAP detected by Fibroscan have been recommended for risk stratification of people with suspected NAFLD [32, 33], such as obesity, we will explore the effectiveness of intervention in obese children with the Fibroscan screen indicators in this study.

This study has several strengths: (1) by randomizing participants at the school level, we avoided the risk of contamination between intervention and control groups; (2) on the basis of a validated school‐based multicomponent intervention approach in the DECIDE-Children study that improved the overall obesogenic environment, in this study, overweight and obese students were additionally treated by professional nutritionists with lifestyle intervention in consideration of personalized characteristics, and interventions were more targeted; and (3) fatty liver indicators were evaluated in this study, as only a few studies with small sample sizes have evaluated the impact of lifestyle intervention on NAFLD [34], and even modest weight loss (> 5%) can produce benefits on NAFLD-related phenotypes [35, 36]. Assessment of the change in fatty liver is meaningful for evaluating the effect of lifestyle intervention; and (4) m-Health technology, including mobile phone apps and sports watches, was applied to strengthen the involvement of caregivers and increase the frequency of participation.

In conclusion, the present study will test the effectiveness of the m-Health-assisted multifaced lifestyle intervention on children with obesity and NAFLD. The results of this study will provide evidence for establishing effective lifestyle intervention strategy aimed at childhood obesity and NAFLD and may help develop guidelines for the treatment of obesity and NAFLD in Chinese children.

### Supplementary Information

**Additional file 1:** SPIRIT 2013 Checklist: Recommended items to address in a clinical trail protocol and related documents.

## Data Availability

Data from the SCIENT study will be made available in the future for collaborative research questions. Such requests must be authorized by the principal investigators and the Ethics Committee of the First Affiliated Hospital of Ningbo University.

## Abbreviations

ALT: Alanine transaminase
BMI: Body mass index
CAP: Controlled attenuation parameters
HDL-c: High-density lipoprotein cholesterol
ICC: Intracluster correlation coefficient
LSM: Liver stiffness measurement
m-Health: Mobile health
NAFLD: Nonalcoholic fatty liver disease
TC: Total cholesterol
TG: Triglyceride
VCTE: Vibration-controlled transient elastography
LDL-c: Low-density lipoprotein cholesterol
