# Supplementary material for: Lifestyle intervention in children with obesity and nonalcoholic fatty liver disease (NAFLD): study protocol for a randomized controlled trial in Ningbo city (the SCIENT study)
Source: Trials. 2024 Mar 20;25:196. doi: 10.1186/s13063-024-08046-4 (PMC10953067; doi:10.1186/s13063-024-08046-4)
Supplement: Supplementary file 1 — Additional file 1: SPIRIT 2013 Checklist: Recommended items to address in a clinical trail protocol and related documents. [file 13063_2024_8046_MOESM1_ESM.doc]

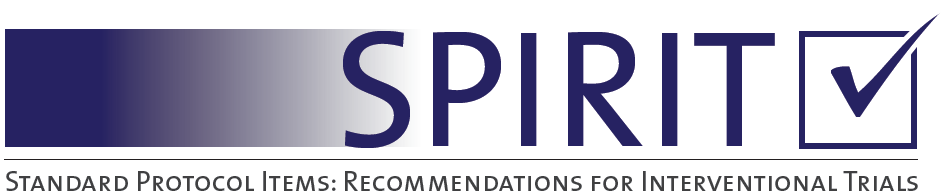


SPIRIT 2013 Checklist: Recommended items to address in a clinical trial protocol and related documents*

| Section/item | Item No | Description | Addressed on page number |
| --- | --- | --- | --- |
| **Administrative information** | | |  |
| Title | 1 | Descriptive title identifying the study design, population, interventions, and, if applicable, trial acronym | 1 |
| Trial registration | 2a | Trial identifier and registry name. If not yet registered, name of intended registry | 4 |
|  | 2b | All items from the World Health Organization Trial Registration Data Set | 4 |
| Protocol version | 3 | Date and version identifier | 2 |
| Funding | 4 | Sources and types of financial, material, and other support | 23 |
| Roles and responsibilities | 5a | Names, affiliations, and roles of protocol contributors | 1, 23 |
|  | 5b | Name and contact information for the trial sponsor | 23 |
|  | 5c | Role of study sponsor and funders, if any, in study design; collection, management, analysis, and interpretation of data; writing of the report; and the decision to submit the report for publication, including whether they will have ultimate authority over any of these activities | 23 |
|  | 5d | Composition, roles, and responsibilities of the coordinating centre, steering committee, endpoint adjudication committee, data management team, and other individuals or groups overseeing the trial, if applicable (see Item 21a for data monitoring committee) | 19 |
| Introduction |  |  |  |
| Background and rationale | 6a | Description of research question and justification for undertaking the trial, including summary of relevant studies (published and unpublished) examining benefits and harms for each intervention | 5-7 |
|  | 6b | Explanation for choice of comparators | 15 |
| Objectives | 7 | Specific objectives or hypotheses | 7 |
| Trial design | 8 | Description of trial design including type of trial (eg, parallel group, crossover, factorial, single group), allocation ratio, and framework (eg, superiority, equivalence, noninferiority, exploratory) | 7 |
| Methods: Participants, interventions, and outcomes | | |  |
| Study setting | 9 | Description of study settings (eg, community clinic, academic hospital) and list of countries where data will be collected. Reference to where list of study sites can be obtained | 7, 8 |
| Eligibility criteria | 10 | Inclusion and exclusion criteria for participants. If applicable, eligibility criteria for study centres and individuals who will perform the interventions (eg, surgeons, psychotherapists) | 8, 9 |
| Interventions | 11a | Interventions for each group with sufficient detail to allow replication, including how and when they will be administered | 9-14 |
|  | 11b | Criteria for discontinuing or modifying allocated interventions for a given trial participant (eg, drug dose change in response to harms, participant request, or improving/worsening disease) | 9 |
|  | 11c | Strategies to improve adherence to intervention protocols, and any procedures for monitoring adherence (eg, drug tablet return, laboratory tests) | 14 |
|  | 11d | Relevant concomitant care and interventions that are permitted or prohibited during the trial | 15 |
| Outcomes | 12 | Primary, secondary, and other outcomes, including the specific measurement variable (eg, systolic blood pressure), analysis metric (eg, change from baseline, final value, time to event), method of aggregation (eg, median, proportion), and time point for each outcome. Explanation of the clinical relevance of chosen efficacy and harm outcomes is strongly recommended | 15, 16 |
| Participant timeline | 13 | Time schedule of enrolment, interventions (including any run-ins and washouts), assessments, and visits for participants. A schematic diagram is highly recommended (see Figure) | 25,26-27 |
| Sample size | 14 | Estimated number of participants needed to achieve study objectives and how it was determined, including clinical and statistical assumptions supporting any sample size calculations | 16 |
| Recruitment | 15 | Strategies for achieving adequate participant enrolment to reach target sample size | 8, 9 |
| **Methods: Assignment of interventions (for controlled trials)** | | |  |
| Allocation: |  |  |  |
| Sequence generation | 16a | Method of generating the allocation sequence (eg, computer-generated random numbers), and list of any factors for stratification. To reduce predictability of a random sequence, details of any planned restriction (eg, blocking) should be provided in a separate document that is unavailable to those who enrol participants or assign interventions | 9 |
| Allocation concealment mechanism | 16b | Mechanism of implementing the allocation sequence (eg, central telephone; sequentially numbered, opaque, sealed envelopes), describing any steps to conceal the sequence until interventions are assigned | 9 |
| Implementation | 16c | Who will generate the allocation sequence, who will enrol participants, and who will assign participants to interventions | 9 |
| Blinding (masking) | 17a | Who will be blinded after assignment to interventions (eg, trial participants, care providers, outcome assessors, data analysts), and how | 7, 9 |
|  | 17b | If blinded, circumstances under which unblinding is permissible, and procedure for revealing a participant’s allocated intervention during the trial | NA: The participants are not blinded with the intervention. |
| **Methods: Data collection, management, and analysis** | | |  |
| Data collection methods | 18a | Plans for assessment and collection of outcome, baseline, and other trial data, including any related processes to promote data quality (eg, duplicate measurements, training of assessors) and a description of study instruments (eg, questionnaires, laboratory tests) along with their reliability and validity, if known. Reference to where data collection forms can be found, if not in the protocol | 15,16, 26, 27 |
|  | 18b | Plans to promote participant retention and complete follow-up, including list of any outcome data to be collected for participants who discontinue or deviate from intervention protocols | 15,16, 27, 28 |
| Data management | 19 | Plans for data entry, coding, security, and storage, including any related processes to promote data quality (eg, double data entry; range checks for data values). Reference to where details of data management procedures can be found, if not in the protocol | 15, 19 |
| Statistical methods | 20a | Statistical methods for analysing primary and secondary outcomes. Reference to where other details of the statistical analysis plan can be found, if not in the protocol | 16, 17 |
|  | 20b | Methods for any additional analyses (eg, subgroup and adjusted analyses) | 17 |
|  | 20c | Definition of analysis population relating to protocol non-adherence (eg, as randomised analysis), and any statistical methods to handle missing data (eg, multiple imputation) | 17 |
| **Methods: Monitoring** | | |  |
| Data monitoring | 21a | Composition of data monitoring committee (DMC); summary of its role and reporting structure; statement of whether it is independent from the sponsor and competing interests; and reference to where further details about its charter can be found, if not in the protocol. Alternatively, an explanation of why a DMC is not needed | 19 |
|  | 21b | Description of any interim analyses and stopping guidelines, including who will have access to these interim results and make the final decision to terminate the trial | 23 |
| Harms | 22 | Plans for collecting, assessing, reporting, and managing solicited and spontaneously reported adverse events and other unintended effects of trial interventions or trial conduct | 14 |
| Auditing | 23 | Frequency and procedures for auditing trial conduct, if any, and whether the process will be independent from investigators and the sponsor | 19 |
| Ethics and dissemination | | |  |
| Research ethics approval | 24 | Plans for seeking research ethics committee/institutional review board (REC/IRB) approval | 22-23 |
| Protocol amendments | 25 | Plans for communicating important protocol modifications (eg, changes to eligibility criteria, outcomes, analyses) to relevant parties (eg, investigators, REC/IRBs, trial participants, trial registries, journals, regulators) | 19 |
| Consent or assent | 26a | Who will obtain informed consent or assent from potential trial participants or authorised surrogates, and how (see Item 32) | 23 |
|  | 26b | Additional consent provisions for collection and use of participant data and biological specimens in ancillary studies, if applicable | 23 |
| Confidentiality | 27 | How personal information about potential and enrolled participants will be collected, shared, and maintained in order to protect confidentiality before, during, and after the trial | 15 |
| Declaration of interests | 28 | Financial and other competing interests for principal investigators for the overall trial and each study site | 23 |
| Access to data | 29 | Statement of who will have access to the final trial dataset, and disclosure of contractual agreements that limit such access for investigators | 24 |
| Ancillary and post-trial care | 30 | Provisions, if any, for ancillary and post-trial care, and for compensation to those who suffer harm from trial participation | NA: The study has no ancillary and post-trial care. |
| Dissemination policy | 31a | Plans for investigators and sponsor to communicate trial results to participants, healthcare professionals, the public, and other relevant groups (eg, via publication, reporting in results databases, or other data sharing arrangements), including any publication restrictions | 18, 24 |
|  | 31b | Authorship eligibility guidelines and any intended use of professional writers | 23 |
|  | 31c | Plans, if any, for granting public access to the full protocol, participant-level dataset, and statistical code | 24 |
| Appendices |  |  |  |
| Informed consent materials | 32 | Model consent form and other related documentation given to participants and authorised surrogates | Attached |
| Biological specimens | 33 | Plans for collection, laboratory evaluation, and storage of biological specimens for genetic or molecular analysis in the current trial and for future use in ancillary studies, if applicable | N/A: The study has no genetic or molecular analysis. |

*It is strongly recommended that this checklist be read in conjunction with the SPIRIT 2013 Explanation & Elaboration for important clarification on the items. Amendments to the protocol should be tracked and dated. The SPIRIT checklist is copyrighted by the SPIRIT Group under the Creative Commons “[Attribution-NonCommercial-NoDerivs 3.0 Unported](http://www.creativecommons.org/licenses/by-nc-nd/3.0/)” license.

**Appendix: Informed Consent Form (Version: 4.0, Date: August 28, 2022)**

Dear Parents,

We invite you and your child to participate in a clinical research study titled "Analysis of the Intervention Effects on Childhood and Adolescent Obesity Based on Structural Equation Modeling and Optimization of Intervention Strategies." Before you and your child decide whether to participate in this study, please carefully read the following information. It will help you understand the study, its purpose, procedures, duration, potential benefits, risks, and inconveniences that may arise from participation. After reading, please decide whether you and your child will participate.

I. Study Introduction

With economic development, improved living standards, and lifestyle changes, the intake of fats and sugars has increased significantly, while physical activity time has decreased, leading to rapid weight gain and an increasing rate of overweight and obesity among children and adolescents in China. This condition not only affects the current health of children but also has implications for their health and quality of life in adulthood.

To promote the healthy growth of children and adolescents, improve students' diet and exercise habits, help students achieve a healthy weight, and enhance physical fitness, the Health Bureau and Education Bureau of Ningbo City, in collaboration with the First Affiliated Hospital of Ningbo University and Professor Wang Haijun's team from the School of Public Health at Peking University, are conducting the Primary School Students' Healthy Weight Management Project in Ningbo. The project is funded by a joint project of the provincial and ministerial levels in Zhejiang Province.

II. Study Process

Who will be invited to participate?

This study will invite all third-grade students from six schools in the districts of Haishu, Yinzhou, and Zhenhai in Ningbo City.

When will it take place?

We will randomly assign schools to the intervention or control group. The intervention group's activities will last for one academic year (September 2022 to June 2023), while the control group will not undergo intervention activities but will receive regular health and physical education. Surveys will be conducted for all third-grade students (including both intervention and control groups) in September 2022 and June 2023.

What are the project contents?

(1) Survey Content

To assess your child's health and evaluate the effectiveness of this activity, we will conduct surveys in September 2022 and June 2023 for all third-grade students (including physical examinations, cardiorespiratory endurance tests, and questionnaires). Physical examinations will include body composition analysis beyond the national routine examination items, fasting blood glucose, and lipid tests (using the remaining blood samples from the national routine student examination, without additional blood collection). The remaining blood samples will only be used for serum studies related to metabolic diseases, metabolomics. The cardiorespiratory endurance test involves a 20-meter shuttle run, conducted by the physical education teacher during physical education classes. Both you and your child will need to complete separate questionnaires to understand your child's diet and physical activity.

If your child is identified as overweight or obese, liver ultrasound examinations will be conducted during the physical examinations in September 2022 and June 2023 to assess the child's fatty liver condition. The survey includes physical examinations (height, weight, waist circumference, hip circumference, body composition analysis, and liver ultrasound), cardiorespiratory endurance tests, and questionnaires.

To observe the long-term effects of intervention on weight maintenance, we will conduct follow-up observations using the fourth, fifth, and sixth-grade national routine examination data for all participating students. Follow-up content includes questionnaires, examination indicators, and cardiorespiratory endurance tests.

(2) Intervention Content

Intervention group schools will carry out the following activities: formulation and implementation of school policies related to obesity, health education activities for students and parents, distribution of health weight knowledge books, and the development of a weight management app for interested parents to learn about weight management health knowledge. If your child is identified as overweight or obese, further in-depth interventions will be implemented: physical education teachers will organize student exercises during double reduction time, monitor student physical activity using free distributed activity bracelets, nutritionists will use the app to manage overweight and obese students, providing dietary, exercise intervention, and health education. Specially designed nutritious meals will be provided by nutritionists for lunch on school days. Students and parents of those not meeting monthly weight loss goals can freely schedule appointments with a multidisciplinary medical team for guidance. During the semester, we will monitor changes in height, weight, waist circumference, hip circumference, and body composition of overweight and obese students and provide timely feedback on weight loss progress.

Control group schools will maintain their existing teaching processes and lifestyles, distributing routine health weight educational materials and not conducting intervention activities. After the intervention group's activities conclude, the control group will receive project-related intervention materials, including policy suggestions, courseware, textbooks, and health education materials, to promote the health status of control group students.

III. Risks and Benefits

Possible Risks and Handling:

During venous blood collection, transient discomfort and/or bruising may occur on the arm, and some individuals may feel faint from needle procedures. If fainting occurs, blood collection will be promptly stopped and properly managed. A brief rest will alleviate the symptoms. In the intervention group, a small number of children may experience minor physical discomfort during sports activities, such as muscle strains, sprains, or pain. If this happens, the physical education teacher will instruct the child to immediately stop exercising, and preliminary checks will be conducted by the school nurse, with hospital referral if necessary.

Direct Benefits to You and Your Child:

All participating students will receive a health examination report, including height, weight, blood glucose, blood lipids, body fat percentage, and other indicators. This allows you to understand your child's health status. The adoption of healthy lifestyle behaviors cultivated during weight management may benefit your child throughout life. Overweight and obese students from the intervention group will also receive a free activity bracelet (to be returned after the project ends) and free weight loss guidance from professional nutritionists.

Potential Benefits:

Your and your child's participation will contribute to a deep analysis of the causes, intervention strategies, and intervention effects of childhood and adolescent obesity from a group perspective. The information obtained from this project will help refine and develop a comprehensive intervention technique for childhood and adolescent weight management.

IV. Fee Explanation

Participation in intervention activities and surveys for children is free of charge.

V. Alternative Options

If you and your child choose not to participate in this study, it will not affect your child's daily learning and life, nor will it cause any medical or other losses to you and your child.

VI. Confidentiality of Subject Information

We will strictly maintain the confidentiality of personal information. The analysis database will not contain any directly identifiable information; instead, coded information will be used for analysis, and the coding information will not be disclosed. We will make every effort to protect the privacy of your and your child's personal medical information within the limits allowed by law.

VII. Subject Rights

In line with the principle of voluntary participation, if you and your child are willing, you can participate in this activity. During your participation in this activity, you can inform us at any time if you and your child wish to withdraw from this activity. Your withdrawal will not affect your child's normal learning and life. After your withdrawal, we will not continue to use or disclose this information.

VIII. Ethics Committee

This study has been reviewed and approved by the Medical Ethics Committee of the First Affiliated Hospital of Ningbo University. For ethical and rights-related matters, you may contact the office of the Medical Ethics Committee of the First Affiliated Hospital of Ningbo University at 0574-87085233. If you have any comments or suggestions regarding this study, you can contact the project personnel Zhang Pingping at the First Affiliated Hospital of Ningbo University, phone: 15071486635.

To facilitate the smooth implementation of this activity, we need the strong support and cooperation of parents. If you and your child agree to participate, please sign below and have your child bring it back to school. You will receive a copy of this informed consent form. Thank you for your cooperation!

I and my child have been informed about the background, purpose, procedures, risks, and benefits of this project and agree to my child's participation.

Student Name: Gender: Grade: Class:

Parent's Signature: Relationship with the Child:

Date: 2022 Month Day

Project Personnel Declaration:

I have informed the parents and children of this project's background, purpose, procedures, risks, benefits, and contact information.

Project Personnel Signature: Contact Information:

Date: 2022 Month: Day:
